# Association of CLDN18 Protein Expression with Clinicopathological Features and Prognosis in Advanced Gastric and Gastroesophageal Junction Adenocarcinomas

**DOI:** 10.3390/jpm11111095

**Published:** 2021-10-26

**Authors:** Antonio Pellino, Stefano Brignola, Erika Riello, Monia Niero, Sabina Murgioni, Maria Guido, Floriana Nappo, Gianluca Businello, Marta Sbaraglia, Francesca Bergamo, Gaya Spolverato, Salvatore Pucciarelli, Stefano Merigliano, Pierluigi Pilati, Francesco Cavallin, Stefano Realdon, Fabio Farinati, Angelo Paolo Dei Tos, Vittorina Zagonel, Sara Lonardi, Fotios Loupakis, Matteo Fassan

**Affiliations:** 1 Oncology Unit 1, Department of Oncology, Veneto Institute of Oncology, Istituto di Ricovero e Cura a Carattere Scientifico (IRCCS), 35128 Padua, Italy; antonio.pellino@iov.veneto.it (A.P.); sabina.murgioni@iov.veneto.it (S.M.); floriana.nappo@iov.veneto.it (F.N.); francesca.bergamo@iov.veneto.it (F.B.); vittorina.zagonel@iov.veneto.it (V.Z.); fotiosloupakis@gmail.com (F.L.); 2 Surgical Pathology Unit, Department of Medicine (DIMED), University of Padua, 35122 Padua, Italy; stefano.brignola@aulss2.veneto.it (S.B.); erika.riello@libero.it (E.R.); mguido@unipd.it (M.G.); gianluca.businello@studenti.unipd.it (G.B.); marta.sbaraglia@aopd.veneto.it (M.S.); angelo.deitos@unipd.it (A.P.D.T.); 3 Department of Pathology, Azienda ULSS 2 Marca Trevigiana, 31100 Treviso, Italy; monia.niero@aulss2.veneto.it; 4 1st Surgery Unit, Department of Surgical, Oncological, and Gastroenterological Sciences (DISCOG), University of Padua, 35122 Padua, Italy; gaya.spolverato@unipd.it (G.S.); puc@unipd.it (S.P.); 5 3rd Surgery Unit, Department of Surgical, Oncological and Gastroenterological Sciences, University of Padua, 35122 Padua, Italy; stefano.merigliano@unipd.it; 6 Surgery Unit, Veneto Institute of Oncology, Istituto di Ricovero e Cura a Carattere Scientifico (IRCCS), 31033 Castelfranco Veneto, Italy; pierluigi.pilati@iov.veneto.it; 7 Independent Statistician, 36020 Solagna, Italy; cescocava@libero.it; 8 Gastroenterology Unit, Veneto Institute of Oncology, Istituto di Ricovero e Cura a Carattere Scientifico (IRCCS), 35128 Padua, Italy; stefano.realdon@iov.veneto.it; 9 Gastroenterology Unit, Department of Surgical, Oncological, and Gastroenterological Sciences (DISCOG), University of Padua, 35122 Padua, Italy; fabio.farinati@unipd.it; 10 Oncology Unit 3, Department of Oncology, Veneto Institute of Oncology, Istituto di Ricovero e Cura a Carattere Scientifico (IRCCS), 35128 Padua, Italy; sara.lonardi@iov.veneto.it; 11 Veneto Institute of Oncology, Istituto di Ricovero e Cura a Carattere Scientifico (IRCCS), 35128 Padua, Italy

**Keywords:** CLDN18.2, gastric adenocarcinoma, biomarkers, immunohistochemistry

## Abstract

**Simple Summary:**

Claudin-18 is a tight junction protein expressed in various cancer types including gastric and gastroesophageal junction cancer. The claudin-18.2 isoform represents a promising target for novel experimental drugs such as zolbetuximab (IMAB362), currently under investigation in several clinical trials of advanced gastrointestinal tumors. In this study, we aim to evaluate the immunohistochemical profile of CLDN18 in a real-world and mono-institutional series of gastric and gastroesophageal carcinomas. The association of CLDN18 expression with clinicopathological features and survival outcomes was investigated.

**Abstract:**

The tight junction protein claudin-18 (CLDN18), is often expressed in various cancer types including gastric (GC) and gastroesophageal adenocarcinomas (GECs). In the last years, the isoform CLDN18.2 emerged as a potential drug target in metastatic GCs, leading to the development of monoclonal antibodies against this protein. CLDN18.2 is the dominant isoform of CLDN18 in normal gastric and gastric cancer tissues. In this work, we evaluated the immunohistochemical (IHC) profile of CLDN18 and its correlation with clinical and histopathological features including p53, E-cadherin, MSH2, MSH6, MLH1, PMS2, HER2, EBER and PD-L1 combined positive score, in a large real-world and mono-institutional series of advanced GCs (*n* = 280) and GECs (*n* = 70). The association of IHC results with survival outcomes was also investigated. High membranous CLDN18 expression (2+ and 3+ intensity ≥75%) was found in 117/350 (33.4%) samples analyzed. CLDN18 expression correlated with age <70 (*p* = 0.0035), positive EBV status (*p* = 0.002), high stage (III, IV) at diagnosis (*p* = 0.003), peritoneal involvement (*p* < 0.001) and lower incidence of liver metastases (*p* = 0.013). CLDN18 did not correlate with overall survival. The predictive value of response of CLDN18 to targeted agents is under investigation in several clinical trials and further studies will be needed to select patients who could benefit from these therapies.

## 1. Introduction

Gastric cancer (GC) still represents a leading cause of cancer death worldwide for both sexes, accounting for over 1 million new diagnoses and thousands of deaths every year [1]. Although multimodal treatments including curative surgery and perioperative/adjuvant chemotherapy have improved outcomes in patients with early-stage disease, the prognosis in those with advanced cancer remains poor [2]. Despite this, the treatment scenario of GC is in continuous evolution [3].

Recent molecular classifications based on gene expression profiling have shed the light on the genomic and epigenomic heterogeneity of this disease and potential predictive and prognostic biomarkers have been identified [4,5,6].

In 2014, The Cancer Genome Atlas (TCGA) research network reported the results of full genomic profiling of 295 primary gastric adenocarcinomas with the identification of four tumor subgroups: microsatellite unstable tumors (22%), Epstein–Barr virus positive (9%), genomically stable tumors (20%) and chromosomally unstable tumors (50%) [7]. Since then, several clinical trials have evaluated immunotherapeutic and targeted agents against metastatic GC. Despite not all have been successful, biological agents have been proven to be a promising tool, alone and in combination with standard treatments [8].

Claudins (CLDNs) are main components of tight junctions that physiologically mediate cell–cell adhesion and regulate selective permeability in epithelial cellular sheets [9]. Particularly, CLDNs are tetraspan proteins ranging from 20 to 27 kDa, composed of four transmembrane domains including a N-terminus and a C-terminus in the cytoplasm and two extracellular loops that span the transmembrane domains [10]. During the last decade, at least 27 CLDN isoforms have been identified in distinct human organs and it has been established that their altered function may play a critical role in tumor carcinogenesis of respective tissues, from metaplasia to progression to metastasis [11,12,13]. In the healthy stomach, CLDNs are confined to gastric mucosa cells (i.e., cells in the pit and base regions of gastric glands) and regulate ion-homeostasis since they act as paracellular proton barriers. Among these, CLDN 1–5, 7–12, 16 and 18 proteins are expressed in normal and healthy gastric mucosa. In particular, the isoform 2 of the tight junction molecule claudin-18 (CLDN18.2), firstly described by Niimi et al. [14], is confined to differentiated and stem gastric epithelial cells where it controls the paracellular permeability to Na+ and H+ ions. Epitopes of CLDN18.2 within the tight junction supramolecular complex are largely inaccessible to intravenous antibodies. However, cell polarity perturbations that occur in the carcinogenesis process lead to exposure of epitopes of CLDN18.2 becoming available for binding of target monoclonal antibodies [14,15,16,17].

In addition, recent studies reported the presence of *CLDN18-ARHGAP* fusions in the genomically stable (GS) GC subtype and a higher frequency of these fusions in diffuse GCs by Lauren classification. The CLDN18-ARHGAP fusion protein may disrupt the structure of the wild-type CLDN18 protein, which may impact the cellular adhesion of cancer cells, increasing their migration and invasion ability. The TCGA data demonstrated that *CLDN18-ARHGAP* fusions were mutually exclusive with *RHOA* and *CDH1* mutations, which were frequently noted in GS-type tumors. Although this molecular feature is one of the molecular characteristics of diffuse GC, the pathogenetic mechanism of the *CLDN18-ARHGAP* fusion gene and its potential targeted therapeutic strategies need further exploration [18].

Moreover, CLDN18.2 expression has been reported in the majority of *CLDN18-ARHGAP* fusion-positive GCs, despite a solid correlation between the presence of *CLDN18-ARHGAP* fusions and CLND18.2 expression has not been demonstrated yet [18,19,20].

Zolbetuximab (IMAB362) is a novel chimeric IgG1 experimental antibody that binds CLDN18.2 on the tumor cell surface and induce immune effectors that activate antibody-dependent cytotoxicity (ADCC) and complement dependent cytotoxicity (CDC) [10]. When combined with chemotherapy, zolbetuximab induces pro-inflammatory cytokines. Final results from the FAST study, an international, multicenter, randomized, phase II trial of epirubicin, oxaliplatin and capecitabine (EOX) with or without zolbetuximab as first-line therapy in patients with advanced GCs and GECs CLDN18.2+, have recently confirmed that addition of zolbetuximab provided a clinically relevant benefit in terms of progression free survival (median 7.9 vs. 4.8 months; HR 0.47; 95% confidence interval (CI) 0.31–0.70, 1-sided *p* = 0.0001), overall survival (median 13.2 vs. 8.4 months; HR 0.51, 95% CI 0.36–0.73, *p* = 0.0001) and overall response rate (43% vs. 28%) compared to EOX alone [17]. Considering these promising results, zolbetuximab is currently under investigation in several phase II and III clinical trials for the treatment of gastrointestinal adenocarcinomas and pancreatic tumors (NCT03504397, NCT03653507 and NCT03816163).

In the last years, a number of retrospective studies have attempted to analyze the clinicopathological characteristics of claudin-18.2-positive GCs/GECs [21,22,23,24]. A recent study from our institution including a large series of 523 primary gastric carcinomas (GCs; *n* = 408) and gastroesophageal carcinomas (GECs; *n* = 115), detected high claudin expression in 29.4% of patients with primary GCs/GECs [25]. According to the results of our study, claudin-18.2 positive membrane CLDN18 expression was statistically associated with non-antral GCs, Lauren diffuse type and with EBV-associated cancers.

Despite emerging clinicopathological features of claudin-18.2 positive GCs/GECs from retrospective works, the relationship between CLDN18.2 expression and its impact on prognosis is still debated and various studies have shown conflicting conclusions [24,26].

For all these reasons, in the present study, we investigated the immunohistochemical profile of CLDN18, and of other biomarkers that are in use in the clinical characterization of GC, such as p53, E-cadherin, MSH2, MSH6, MLH1, PMS2, HER2, EBER and PDL-1 combined positive score (CPS) in a large series of locally advanced or metastatic GCs (*n* = 280) and GECs (*n* = 70), focusing on the association of CLDN 18 expression with IHC results and survival outcomes.

## 2. Materials and Methods

### 2.1. Case Selection

Medical records of patients diagnosed with advanced GC and GEC referred to the Unit of Medical Oncology 1 of the Veneto Institute of Oncology (IOV-IRCCS) of Padua, from January 2010 to July 2019 were retrospectively selected, and then prospectively followed until January 2020. The study was approved by our Institutional Review Board, and was included in the observational retrospective study “GAS-ALL-IN—GAStric cancers a retrospective analysis of ALL major prognostic and predictive determINants”.

A total of 435 samples obtained from 366 patients with adequate clinical information constituted our clinical series. Clinicopathological parameters collected were the following: sex, age at diagnosis, date of first diagnosis of GC, site of origin (GC vs. GEC), histotype, tumor grading, immunohistochemical profile of CLDN18, p53, E-cadherin, MSH2, MSH6, MLH1, PMS2, HER2, EBER, PDL-1 combined positive score (CPS), Lauren, TCGA, Asian Cancer Research Group (ACRG), Ahn [27] and Birkman [28] classifications and overall survival (OS).

All cases with available tissue samples were re-evaluated by two gastrointestinal pathologists to assess the quality of samples for immunohistochemical analyses, to confirm the primary tumor site and pathological characteristics [29,30]. The tissues were collected and processed similarly according to standard protocols, with formalin fixation time <48 h. Twenty-two samples (5.1%) resulted inadequate for immunostaining due to tumor exhaustion, which corresponded to 16 patients who were excluded. Thus, 350 patients and their corresponding 413 samples were eligible and constituted our dataset.

### 2.2. Immunohistochemistry (IHC)

IHC was performed by using: (I) the OptiView DAB IHC Detection Kit in the BenchMark IHC/ISH autostainer (Roche Ventana Medical Systems, Oro Valley, AZ, USA) with the antibody for CLDN18 (clone 43-14A; Roche Ventana, Oro Valley, AZ, USA); (II) using the Bond Polymer Refine Detection kit (Leica Biosystems, Newcastle upon Tyne, UK) in the BOND-MAX system (Leica Biosystems) with the primary antibodies for proteins of the DNA mismatch repair complex (MLH1 (ES05; Dako, Carpinteria, CA, USA), MSH2 (FE11; Dako, Carpinteria, CA, USA), MSH6 (EP49; Dako), and PMS2 (EP51; Dako)), p53 (clone DO-7; Dako, Carpinteria, CA, USA), Oracle HER2 Bond IHC system (clone CB11; Menarini Diagnostics, Florence, Italy), E-cadherin (clone NCH-38; Dako, Carpinteria, CA, USA) and PDL-1 (clone 22C3; Dako, Carpinteria, CA, USA). All the slides were jointly evaluated by two pathologists.

*CLDN18-ARHGAP* fusion gene is highly enriched in the GS subtype, and the clinical and prognostic meaning of the *CLDN18-ARHGAP* fusion in GC has been recently investigated. However, considering that data about the fusion mostly derive from retrospective studies, with various detection methods and limited follow-up duration at the time of drafting the protocol, we did not include *CLDN18-ARHGAP* analysis other than CLDN18 membrane immunoreaction, currently used in ongoing zolbetuximab studies.

For the evaluation of CLDN18, the membrane immunoreaction was assessed according to manufacturer’s protocol after a web-based training (Roche Ventana, Oro Valley, AZ, USA). Both the intensity of tumor cell membrane staining and the percentage of cancer cells with complete, basolateral or lateral membrane staining were assessed. Only the percentage of tumor cells with strong to moderate (2+/3+) staining intensity confirmed by immunohistochemistry in a formalin-fixed paraffin-embedded (FFPE) tumor sample were retained for scoring. Lung samples and already known CLDN18 positive GCs [25] were used as positive controls. The whole tumor section was considered for the scoring. Cases were dichotomized according to a cut-off of 2+ or 3+ intensity in ≥75% of the tumor cells, which is the IHC cut-off being used for eligibility in ongoing zolbetuximab studies.

Deficient mismatch repair (MMRd) status was assessed by testing MSH2, MSH6, MLH1 and PMS2, and samples were defined as MMRd when PMS2 or MSH6 or both proteins of the heterodimer resulted negative [31].

P53 was considered as aberrant in the presence of complete loss or diffuse and strong nuclear immunostaining in neoplastic cells [25].

For the evaluation of HER2, the four-tier score was adopted [31]; equivocal cases were analyzed by HER2 chromogenic in situ hybridization (CISH) to test for gene amplification.

E-cadherin expression was considered altered in the presence of complete loss or markedly reduced membranous staining (>30%), regardless of nuclear/cytoplasmic staining.

PD-L1 expression was assessed as CPS (combined positive score) according to manufacturer’s protocols [32].

### 2.3. HER2 Chromogenic In Situ Hybridization (CISH)

CISH was performed according to the manufacturer’s protocol (Dako Her2 CISH pharmDx Kit; Dako, Glostrup, Denmark). Areas with the highest HER2 counts with non-overlapping nuclei were analyzed by counting HER2 and centromeric probe 17 (CEP17) signals in at least 40 nuclei. The ratio HER2/CEP17 was calculated. A case was considered HER2 amplified when the signal ratio was ≥2.0 or when HER2 signal cluster was observed.

### 2.4. EBER In Situ Hybridization (ISH)

The Bond ready-to-use ISH EBER Probe was used in a Leica Bond-Max automation system according to the manufacturer’s instructions (Leica Biosystems, Wetzlar, Germany) to detect EBV infection.

### 2.5. IHC Profiling for Molecular Classification

Immunohistochemical profiling has recently emerged as a suitable alternative for molecular classification of GC. We focused on the work of Ahn [27] and Birkman [28] to IHC categorize our series according to TCGA and ACRG molecular classifications of GC.

### 2.6. Construction of Virtual Biopsies

Tumor heterogeneity has been observed to be extremely relevant in GCs/GECs. An accurate assessment of specific biomarkers status is necessary to select patients who may benefit from targeted agents. Regarding HER2 testing, problematic issues include pre-analytic variables, the identification of the best tissue samples to perform HER2 analysis, heterogeneity within the primary tumor and heterogeneity between primary tumor and metastases. The mechanisms leading to HER2 expression heterogeneity are still largely unknown, but possibilities include neoplastic clones in which HER2 is amplified/overexpressed in an otherwise HER2 negative tumor or silencing of HER2 expression in an area of a tumor with homogeneous HER2 amplification [33]. In order to improve the accuracy of HER2 testing, comprehensive guidelines for the assessment of HER2 in patients with GCs/GECs have been established [34]. Our group has recently investigated intratumoral variability of membranous CLDN18 expression in a large series of GCs/GECs samples [25]. A tumor was considered as CLDN18 heterogeneous in case of concomitant presence of high-CLDN18 and low-CLDN18 tissue microarray cores. Among primary tumors, 160 GCs (40.3%) and 38 GECs (33.6%) showed intratumoral variability within the analyzed cores. Similar results were observed for metastatic samples. However, CLDN18 status was concordant between primary and metastatic samples in 111 cases (86.7%; 83 negative and 28 positive cases), with only 17 cases showing CLDN18 discordant status.

To further investigate CLDN18 intratumor heterogeneity, we performed a virtual biopsy study in 93 surgically treated cases (77 GCs, 16 GECs), staining two separate FFPE blocks for CLDN18, digitally scanned (Aperio ScanScope XT system; Leica Biosystems, Wetzlar, Germany) and digitally saved with an “.svs” extension. A dedicated Ellipse tool was used to select circular areas, corresponding to “virtual biopsies” on both of the two surgical specimen slides selected for every case. These areas were 2.6 mm in diameter, which is estimated to be the average diameter of endoscopic gastric biopsies [35].

The selected virtual biopsy area was drawn on the luminal part of the sample, thus simulating superficial biopsy samples obtained at endoscopy. This evaluation was only retained for the virtual biopsy study, otherwise the whole tumor section was evaluated for CLDN18 expression. In order to prevent selection bias (secondary to the selection of IHC-positive areas during the virtual sampling process), the virtual biopsies were outlined by a person with no diagnostic experience and not aware of the study purpose. Circles were used to outline 5 randomly spaced virtual biopsy areas on the luminal surface of the digital slide.

A total of 10 virtual biopsies (5 on each of the 2 digital slides available for each case) were selected for each tumor. For each virtual biopsy, the CLDN18 IHC evaluation was performed by two gastrointestinal pathologists blinded to the CLDN18 status in the rest of the slide. The percentage of 2+/3+ CLDN18 positive tumor cells in each virtual biopsy was evaluated.

### 2.7. Statistical Analysis

Statistical analysis was performed using R 4.1 (R Foundation for Statistical Computing, Vienna, Austria). Associations between CLDN18 and clinicopathological variables or other IHC markers were investigated the χ^2^ test and Fisher Exact test. No multiplicity correction procedure was planned and a *p* value < 0.05 was considered statistically significant. Survival curves were calculated using the Kaplan–Meier method and compared using the log-rank test.

## 3. Results

### 3.1. Clinical Characteristics

Overall, the median age of patients at diagnosis of metastatic disease was 66 years (range 28–92), 218 were male (62.3%) and 132 were female (37.7%) (Table 1). All patients were Caucasian. These characteristics were comparable to those of the initial population of 366 patients. Altogether, 182 patients (52.0%) presented a Performance Status (PS) ECOG 0 at diagnosis of metastatic disease, and 168 patients (48.0%) a PS ≥ 1. Primary tumor site was stomach in 280 patients (80.0%) and gastro-esophageal junction in 70 patients (20.0%). Primary tumor was resected in 185 patients (52.9%), margins were R0 in 126 patients (36.0%), R1/R2 in 32 patients (9.1%). TNM stage at diagnosis, according to the American Joint Cancer Committee/Union for International Cancer Control (AJCC/UICC) 8th edition was I in 5 patients (1.4%), II in 32 patients (9.2%), III in 89 patients (25.4%) and IV in 210 patients (60.0%). Metastasis were synchronous in 253 patients (72.3%) and metachronous in 97 patients (27.7%); 144 patients (41.1%) presented more than one site of metastatic disease at diagnosis. The most frequent metastatic site was peritoneum (146; 41.7%); 142 patients (40.3%) had nodal involvement and 35 patients (10.0%) had lung localizations.

### 3.2. Histopathological Characteristics

Primary samples were 309 (88.3%), 245 (70.0%) from GCs and 64 (18.2%) from GECs while the metastatic samples were 41 (11.7%), 35 from GCs (10.0%) and 6 from GECs (1.7%). According to Lauren’s criteria, 117 cases were diffuse type (33.4%), 186 intestinal type (53.1%) and 47 (13.4%) were indeterminate subtypes (mixed or rare). Considering the 2019 World Health Organization criteria [36], among the adenocarcinomas 180 (51.0%) were tubular, 65 (18.6%) were poorly cohesive carcinomas including 52 signet-ring type; (14.9%), 39 (11.4%) were mixed and 14 (4.0%) presented a rare histological subtype. When evaluating the grade of differentiation, 13 cases (2.0%) were G1, 83 cases (23.7%) were G2, 84 cases (24.0%) were G3 and 170 cases (48.57%) were not evaluable.

A total of 52 (14.9%) tumors showed a HER2 overexpression or HER2 gene amplification (Table 2). Mismatch repair deficiency was observed in 54 cases (15.4%) and a total of 8 (2.3%) tumors were positive for EBER ISH. According to PD-L1 expression, a Combined Positive Score (CPS) ≥ 1 was found in 98 cases (28%); 71 patients (20.3%) presented a CPS ≥ 5. Eighty (23.0%) samples showed completely loss of E-cadherin membranous expression, while p53 alterations were present in 168 (48.0%) of the cases.

As reported in Table 3 samples were classified according to Lauren, TCGA, Asian Cancer Research Group (ACRG), Birkman and Ahn classification according to their immunohistochemical profile.

### 3.3. CLDN18 Expression, Prevalence and Clinicopathological Associations

In cancer cells, CLDN18 was considered as positive only if membranous staining was present. Any CLDN18 expression (2+ and 3+ intensity in 1–100% tumor cells) was reported in 261/350 (70.6%) patients (234 primary cases, 27 metastases). However, high expression levels of CLDN18 (2+ and 3+ intensity in ≥75% tumor cells) were found in 117/350 (33.4%) patients (98 primary cases, 19 metastases) and 158/350 (45.1%) patients presented at least 50% of the cancer cells stained for CLDN18.

In 27 cases, matched samples from primary and metastatic tumors were available; in 27 cases, two different samples (biopsy and surgical) of the primary tumor were analyzed. In 9 cases, a third tumor sample was analyzed. Among the 27 cases with two tumor samples available, 18 (66.7%) cases showed concordance in CLDN18 status (13 negative and 5 positive) and 9 were discordant. Among the 27 cases with matched primary and metastatic samples, 22 (81.5%) showed concordance in CLDN18 status (16 negative and 6 positive), and 5 were discordant (1 case with a positive primary tumor and a negative metastasis); 4 cases with a positive metastatic sample in the presence of a negative primary tumor sample.

**Figure 1:**
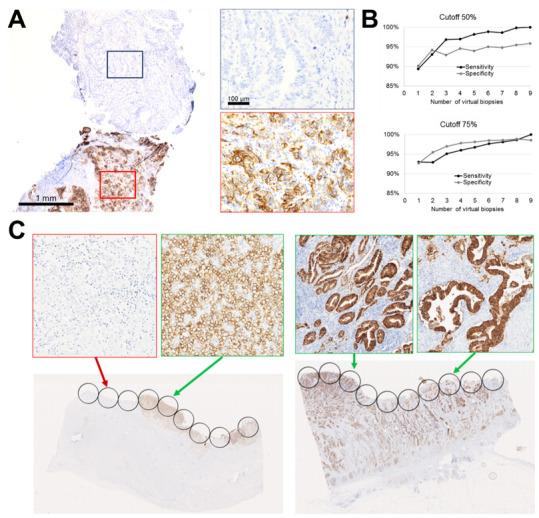
(**A**) Representative example of intratumor CLDN18 heterogeneity; two biopsies from the same primary cancer show dissimilar CLDN18 status. (**B**) Sensitivity and specificity of CLDN18 testing according to the number of virtual biopsies scored. (**C**) Two examples of a heterogeneous CLDN18 tumor (left panel) and a gastric adenocarcinoma with a homogeneous CLDN18 status in all the analyzed areas. The selected virtual biopsy area was drawn on the luminal part of the sample, thus simulating superficial biopsy samples obtained at endoscopy. (Original magnifications 1×, 5× and 20×).

To further dissect CLDN18 intratumor heterogeneity (Figure 1), we performed a virtual biopsy study staining two separate FFPE blocks of 93 surgically treated GCs/GECs. Sensitivity increased from 2 to 9 biopsies (93–100%); after 6 biopsies, the increase was more contained: 97.7–98.2% (from 6 to 7 biopsies), 98.2–98.7% (from 7 to 8 biopsies) and 98.7–100% (from 8 to 9 biopsies). It must be underlined that considering 2 to 3 biopsies gives a sensitivity of 92.9–95.1% in adequately assessing CLDN18 status. Specificity was stable between 6–8 biopsies (98.5–98.9%). Overall, these data support, as already observed for other predictive biomarkers in GCs, that, if possible, CLDN18 status should be assessed in multiple biopsies obtained from the primary tumor to overcome intratumor heterogeneity, and that 6 biopsies should be considered as a standard of sampling for an adequate immunohistochemical profiling. This was more evident when considering a cut-off of 50%.

**Figure 2:**
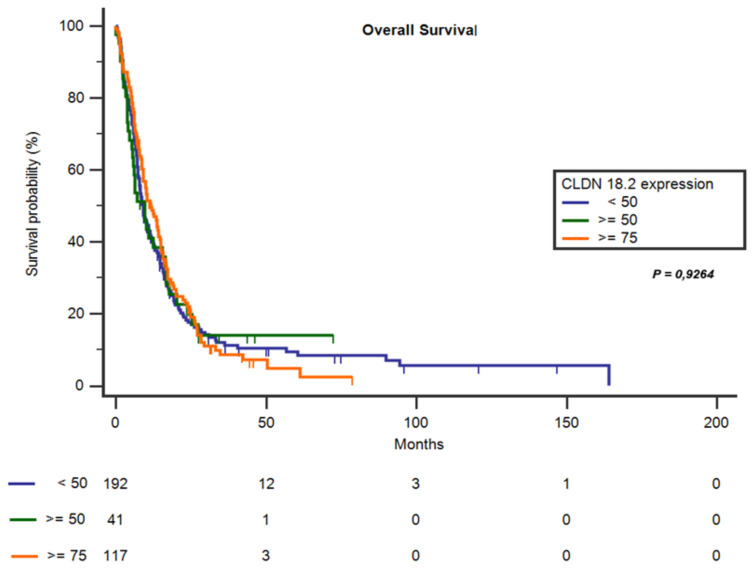
Overall survival time in patients with CLDN18 expression (cut-off of 75% and 50%) compared with patients who were claudin-18 negative (<50%).

The main clinical characteristics in the subgroups of altered CLDN18 (≥75%) are described in Table 1. CLDN18 expression correlated with nodal involvement (*p* = 0.0407), high stage disease (III, IV) at diagnosis (*p* = 0.019), age < 70 (*p* = 0.0035), peritoneal involvement (*p* < 0.001) and lower incidence of liver metastases (*p* = 0.009). No other significant differences between CLDN18 expression and clinicopathological data were observed. Expression of CLDN18 at different cut-offs (<50%; ≥50%; ≥75%) was not associated with a shortened or prolonged OS time (*p* = 0.9264) in patients with GCs and GECs (Figure 2).

A significant association was observed between CLDN18 and EBV status (*p* < 0.001) (Table 2). Particularly, high expression levels of CLDN18 were identified in 7/8 (87.5%) of EBER positive tumors. All of them were GCs. The mean age of EBER positive patients was 59.2 years of which 7/8 (87.5%) were male. No association emerged between CLDN18 expression and p53, HER2, E-cadherin, PD-L1 (CPS ≥ 1 and CPS ≥ 5) or MMRd.

As reported in the work of Ahn et al. [27], we reproduced TCGA and ACRG molecular classifications, using immunohistochemical analysis (Table 3). According to TCGA molecular subtype 220 cases were CIN subtype (74 CLDN18.2 high; 33.6%), 65 were GS (21 CLDN18.2 high; 32.3%), 54 were MSI (15 CLDN18.2 high; 27.7%) and 8 were EBV subtype (7 CLDN18.2 high; 87.5%). According to ACRG molecular classification 54 were MSI (15 CLDN18.2 high; 27.7%), 121 MSS/p53-(35 CLDN18.2 high; 28.9%), 105 MSS/p53 + (43 CLDN18.2 high; 41%) and 68 MSS/EMT (24 CLDN18.2 high; 35.3%). No significant association has been observed between CLDN18 expression and all these classifications, but the presence of EBV infection.

## 4. Discussion

In the last years, a better understanding of molecular mechanisms involved in GC pathogenesis has led to the development of new targeted approaches. Unfortunately, at the present time, only a few targeted therapies have demonstrated to improve the outcome of patients with advanced GC. The isoform 2 of claudin-18 protein, located on the outer cell membrane in a significant percentage of GCs, represents a possible target for monoclonal antibody such as zolbetuximab (IMAB362), currently under investigation in several gastrointestinal clinical trials [37]. Considering the emergent role of these agents, the aim of this study was to investigate the immunohistochemical expression of claudin-18 in a large real-world series of advanced GCs and GECs and correlate these IHC results with clinicopathological features and survival outcomes.

In our analysis, positivity was defined as ≥2+ and 3+ CLDN 18 staining intensity in ≥75% of tumor cells. Notably, the cut-off value of CLDN18 expression varies across different studies. In the FAST phase II study, a trial of epirubicin, oxaliplatin and capecitabine (EOX) with or without the anti-CLDN18.2 antibody zolbetuximab, patients were eligible if their tumors had ≥40% of tumor cells expressing CLDN18.2 with a moderate-to-strong (≥2+) staining intensity using the CLAUDETECT™18.2 test and the 43-14A antibody clone [17]. Moreover, preplanned analyses from the FAST trial showed that higher levels of expression of CLDN18 (≥2+ in ≥70% of tumor cells) achieved a greater treatment advantage of zolbetuximab than lower levels. Ongoing trials (NCT03505320, NCT03504397) are evaluating the efficacy of zolbetuximab in patients with staining intensity in ≥75% of tumor cells using the Ventana test which has been shown to be equivalent to the 70% cut-off used with the manual test used in the FAST clinical study. For all these reasons, our cases were dichotomized according to the Ventana test using a cut-off of ≥75%, which is the IHC cut-off being used for eligibility in ongoing zolbetuximab studies.

A significant proportion of positive GCs and GECs from our dataset showed low prevalence of CLDN18 staining (2+ and 3+ intensity in 1–49% of tumor cells) while high CLDN18 expression (2+ and 3+ intensity in ≥50% of tumor cells) was found in 158/350 (45.1%) cases and 117/350 (33.4%) patients presented at least 75% of the cancer cells stained for CLDN18. These findings are consistent with previous results, although as mentioned above, there are divergences of CLDN18 expression rates across studies. In the MONO trial moderate or strong (2+/3+) CLDN18 membrane staining intensity in 50% of tumor cells was reported in 54/120 patients (45%) and higher expression levels (2+/3+ in 70% of tumor cells) appeared in 29/120 (24%) of patients [38]. In the FAST study CLDN18 expression (≥2+ intensity in ≥40% tumor cells) was found in 334/686 patients (48%) and 249/686 (36.3%) patients had 2+/3+ CLDN18 staining in ≥70% of tumor cells [17]. In a Japanese retrospective study, using the same diagnostic antibody we used in our series (i.e., 43-14A), moderate-to-strong CLDN18 expression (eligibility criterion in FAST) was observed in 135/262 (52%) of primary tumors and 61/135 (45%) of nodal metastases [21]. This divergence of CLDN18 expression rates can be explained by different ethnic characteristics of the cohorts, intratumoral variability, heterogeneity of CLDN18 expression and limitations of small tissue specimens. However, most of them should be related to the use of different antibodies to detect CLDN18 expression. Hence, defining standard immunostaining and scoring protocols will be crucial for the generation of comparable data in future studies. According to our findings, about one third of advanced GCs/GECs exhibit high CLDN18 expression levels (≥2+ in ≥75% of tumor cells) with potential implications for the near clinical practice. If the ongoing phase III trials (SPOTLIGHT, GLOW) will successfully elucidate the true benefit of zolbetuximab, CLDN18 could be approved as a predictive biomarker in GCs/GECs. However, considering the recent data of survival benefit with upfront nivolumab in combination with platinum-based chemotherapy that will shift the standard of care in advanced GCs/GECs, more studies will be needed to clarify how to choose between checkpoint vs. CLDN18 blockade.

Destruction of tight junctions during tumorigenesis leads to disruption of epithelial cell cohesion, contributing to cell invasiveness and formation of metastases [39,40]. Various previous studies suggested lower expression levels of claudin proteins in tumor cells as a characteristic of GC [12,23,24,41,42]. In our cohort, 89 (25.4%) specimens were completely devoid of any CLDN18 expression and 18 (5.1%) presented only a weak (IHC 1+) staining. In line with previous works, in the present study data from stage I and II GC (Table 1) are highly suggestive for a down-regulation of claudin-18 that occurs early in process of tumorigenesis. However, high CLDN18 expression is maintained in stage III, IV disease (*p* = 0.019) and 158/350 (45%) samples presented at least 50% of the cancer cells stained with ≥2+ intensity for CLDN18. Further studies are needed to clarify the hypothesis of CLDN18 downregulation/expression as a characteristic of tumorigenesis in GC progression.

We observed a statistically significant higher expression of CLDN18 in patients diagnosed before 70 years old, with high stage disease (III, IV) at diagnosis, peritoneal involvement and lower incidence of liver metastases. No other significant association emerged in relation to clinicopathological characteristics (sex, gastric vs. gastro-esophageal tumor localization, grading) and Lauren, TCGA, ACRG and Birkman and Ahn classifications. Although previous works showed a higher percentage of CLDN18 prevalence in diffuse/poorly cohesive GC type, we did not find any association with Lauren classification [17]. Our group previously demonstrated a correlation between positive CLDN18 cases and diffuse type GC, however in our previous series only the 8.6% of cases were stage IV tumors and this can significantly affect the prevalence of CLDN18-positive cases [25]. Consistent with the present results, a recent study from a large Caucasian cohort did not find correlation with Lauren phenotype [22].

Previous studies have found that CLDN18.2 and E-cadherin expression were decreased in patients with peritoneal metastases compared to those without peritoneal involvement [24,41,43]. Interestingly, in our cohort CLDN18 is well preserved in patients with peritoneal metastases. To date, the precise mechanism of interaction between CLDN18.2 expression and specific metastatic spread is not well defined and further studies investigating these mechanisms are needed.

No association was found between CLDN18 expression and p53, HER2, E-cadherin, PD-L1 (CPS ≥ 1 and CPS ≥ 5) or MMRd. Consistent with previous results, CLDN18-positive predominance in EBV-associated GCs was confirmed in our cohort. Taking into account the small sample size, high expression levels of CLDN18 were identified in 7/8 (87.5%) of EBER positive tumors. The preserved expression of CLDN18 in EBV gastric tumor cells is probably linked to key features of EBV-mediated tumorigenesis. EBER positive GCs are a unique etiological entity with specific characteristics such as predominance among males and a predominant location in the proximal stomach [44,45]. Recent data suggest that overexpression of programmed death ligands 1 and 2 (PD-L1 and PD-L2) and of a significant immune cell presence in EBV GCs could support the rationale to evaluate checkpoint inhibitors in this GC subgroup [46]. Among the 8 EBV positive samples, a large proportion (75%) showed a CPS ≥ 1, confirming a significant expression of PD-L1 in EBV positive GCs microenvironment.

In our dataset, CLDN18 expression did not correlate with overall survival. Two previous studies from a 134-patient cohort [12] and a 65-patient cohort [23] with advanced GCs suggested that reduced CLDN18.2 expression correlates with poor prognosis. Consistent with our findings, recent works have confirmed that CLDN18.2 expression did not impact on prognosis of GC and GECs [22,26].

In routine practice, CLND18 assessment will rely on endoscopy biopsies from the primary tumor. Nonetheless, intrinsic CLDN18 expression heterogeneity has been observed in some cases. Our virtual biopsy study supports the use of at least six biopsies to adequately assess CLND18 expression, which is in line for what is performed and has been demonstrated for HER2 testing in GCs/GECs [31]. However, high diagnostic performances were also observed with a lower number of biopsies. The high concordance rate between metastatic site and primary tumor indicates that accurate testing can be performed from both primary GC and metastasis.

A limitation of this study is that all tumor samples were collected from one single institution (around 1.5 million people in the catchment area), so that these data may not accurately represent the overall patient population. Despite this, the large cohort size (350), together with the long timespan over which these samples and clinical data were collected (January 2010 to July 2019), could reduce the risk of inaccuracy in the final results. Another limitation is the lack of analyses corrected for multiple testing. However, no significant association between claudin-18.2 and clinico-pathological parameters emerged in our study, which is exploratory and “hypotheses-generating”. We maintained the direct correlations between parameters, as considered in the primary plan of the study. Moreover, CLDN18 expression results are very similar to those observed in other Caucasian and Japanese populations. Any difference between Caucasian and Asian cohorts will be hopefully elucidated by the results of the international ongoing trials.

## 5. Conclusions

In this study, we provide a detailed description of CLDN18 expression and its correlation with clinicopathological characteristics and survival outcomes in a large series of locally advanced and metastatic GCs and GECs. Previous studies have shown conflicting conclusions and it has not been clarified yet whether CLDN18.2 have a prognostic impact. In the present work CLDN18 expression does not correlate with prognosis and further investigations are needed to explore and validate more prognostic predictors of CLDN18.2. At the present time, ongoing phase III trials are comparing combinations of chemotherapy plus zolbetuximab as first-line treatment in CLDN18.2-enriched patient populations (NCT03504397 and NCT03653507). Recent FDA immunotherapy approvals for the initial treatment of patients with advanced GC/GECs have expanded the treatment options for patients with GC/GECs (https://www.fda.gov/news-events/press-announcements/fda-approves-first-immunotherapy-initial-treatment-gastric-cancer; accessed on 15 August 2021). Therefore, results from prospective studies are warranted to better select patients who could benefit from anti-CLDN18.2 therapies and combining different strategies may further improve the outcome of these patients. Stepping in this direction, a phase II study of zolbetuximab plus pembrolizumab in claudin-18.2 positive GC/GECs has been initiated (NCT03505320). In this scenario, CLDN18.2 represents a promising target among HER2 negative GC/GECs and could emerge as an important predictive biomarker, together with HER2, in the treatment of GC/GECs.

## Figures and Tables

**Table 1:** Clinicopathological features of the considered series according to CLDN18 status (note that the sum of patients does not add to 350 patients for all the parameters due to missing clinical data or exhausting tumor tissue).

Patients’ Characteristics		Total 350 *n*. (%)	CLDN18 < 75% Tot 233 *n*. (% of the Total)	CLDN18 ≥ 75% Tot 117 *n*. (% of the Total)	*p* Value
Sex	Male	218 (62.3)	138 (39.4)	80 (22.9)	0.1214
	Female	132 (37.7)	95 (27.1)	37 (10.6)	
Age	<70	209 (59.7)	126 (36.0)	83 (23.7)	**0.0035**
	>70	141 (40.3)	107 (30.6)	34 (9.7)	
ECOG PS	0	176 (51.2)	111 (32.3)	65 (18.9)	0.1571
	≥1	168 (48.8)	119 (34.6)	49 (14.2)	
Primary resection	Yes	185 (52.9)	126 (36.0)	59 (16.9)	0.5949
	No	165 (47.1)	107 (30.6)	58 (16.6)	
pT	1–2	18 (5.1)	14 (4.0)	4 (1.1)	0.4325
	3–4	136 (38.9)	93 (26.6)	43 (12.3)	
	Missing	196 (6)	126 (36.0)	70 (20.0)	
pN	0–1	54 (15.4)	44 (12.6)	10 (2.9)	**0.0407**
	2–3	98 (28.0)	62 (17.7)	36 (10.3)	
	Missing	198 (56.57)	127 (36.3)	71 (20.3)	
Stage at first tumor diagnosis	I–II	37 (10.6)	33 (9.4)	4 (1.1)	**0.0033**
	III–IV	299 (85.4)	192 (54.9)	107 (30.6)	
	Missing	14 (4.0)	8 (2.3)	6 (1.7)	
Metastatic disease	Synchronous	253 (72.3)	163 (46.6)	90 (25.7)	0.2124
	Metachronous	97 (27.7)	70 (20.0)	27 (7.7)	
Sites of metastases at baseline	liver yes	132 (37.7)	99 (28.3)	33 (9.4)	**0.0130**
	liver no	218 (62.3)	134 (38.3)	84 (2)	
	nodes yes	141 (40.3)	95 (27.1)	46 (13.1)	0.8835
	nodes no	209 (59.7)	138 (39.4)	71 (20.3)	
	lung yes	35 (10.0)	26 (7.4)	9 (2.6)	0.4060
	lung no	315 (90.0)	207 (59.1)	108 (30.9)	
	peritoneum yes	146 (41.7)	79 (22.6)	67 (19.1)	**<0.001**
	peritoneum no	204 (58.3)	154 (44.0)	50 (14.3)	
	bone yes	30 (8.6)	17 (4.9)	13 (3.7)	0.3172
	bone no	320 (91.4)	216 (61.7)	104 (29.7)	
	brain yes	5 (1.4)	4 (1.1)	1 (0.3)	0.8700
	brain no	345 (98.6)	229 (65.4)	116 (33.1)	
	other yes	33 (9.4)	22 (6.3)	11 (3.1)	1.000
	other no	317 (90.6)	211 (60.3)	106 (30.3)	
Location	G	280 (80.0)	190 (54.3)	90 (25.7)	0.3799
	GEJ	70 (20.0)	43 (12.3)	27 (7.7)	
Histology	Mixed YES	39 (11.1)	25 (7.1)	14 (4.0)	0.8676
	Mixed NO	311 (88.9)	208 (59.4)	103 (29.4)	
	PCC YES	65 (18.6)	42 (1)	23 (6.6)	0.8222
	PCC NO	285 (81.4)	191 (54.6)	94 (26.9)	
	PCC–signet ring YES	52 (14.9)	28 (8.0)	24 (6.9)	0.051
	PCC–signet ring NO	298 (85.1)	205 (58.6)	93 (26.6)	
	Rare YES	14 (4.0)	9 (2.6)	5 (1.4)	1.000
	Rare NO	336 (96.0)	224 (6)	112 (3)	
	Tubular YES	180 (51.4)	129 (36.9)	51 (14.6)	0.0500
	Tubular NO	170 (48.6)	104 (29.7)	66 (18.9)	
Grading	G1	13 (3.7)	8 (2.3)	5 (1.4)	0.0834
	G2-G3	167 (47.7)	121 (34.6)	46 (13.1)	
	NE	170 (48.6)	104 (29.7)	66 (18.9)	
Lauren	Diffuse YES	117 (33.4)	70 (20.0)	47 (13.4)	0.0760
	No	233 (66.6)	163 (46.6)	70 (20.0)	
	Intestinal YES	186 (53.1)	132 (37.7)	54 (15.4)	0.0813
	NO	164 (46.9)	101 (28.9)	63 (18.0)	
	Mixed YES	39 (11.1)	25 (7.1)	14 (4.0)	0.8676
	NO	311 (88.9)	208 (59.4)	103 (29.4)	

**Table 2:** Immunohistochemical profiling of the considered series according to CLDN18 status (note that the sum of patients does not add to 350 patients for all the parameters due to missing clinical data or exhausting tumor tissue).

Patients’ Characteristics		Total 350 *n*. (%)	CLDN18 < 75% Tot 233 *n*. (% of the Total)	CLDN18 ≥ 75% Tot 117 *n*. (%of the Total)	*p* Value
MMRd	Yes	54 (15.4)	39 (11.1)	15 (4.3)	0.2424
	No	296 (84.6)	194 (55.4)	102 (29.1)	
HER 2 status	Positive	52 (14.9)	35 (10.0)	17 (4.9)	1.000
	Negative	298 (85.1)	198 (56.6)	100 (28.6)	
PD-L1 CPS ≥ 1	Yes	98 (28)	68 (19.4)	30 (8.6)	0.5685
	No	252 (72)	165 (47.14)	87 (24.86)	
PD-L1 CPS ≥ 5	Yes	71 (20.29)	50 (14.29)	21 (6)	0.5290
	No	279 (79.71)	183 (52.29)	96 (27.43)	
EBER	Positive	8 (2.3)	1 (0.3)	7 (20.0)	**0.0024**
	Negative	342 (97.7)	232 (66.3)	110 (31.4)	
p53 status	Altered	168 (48.0)	111 (31.7)	57 (16.3)	0.9676
	wild type	181 (52.0)	121 (34.6)	60 (17.1)	
E-Cadheri *n* status	Positive	268 (77.0)	177 (50.9)	91 (26.1)	0.9148
	Negative	80 (23.0)	54 (15.5)	26 (7.5)	

**Table 3:** Immunohistochemical molecular classification of the considered series according to CLDN18 status (note that the sum of patients does not add to 350 patients for all the parameters due to missing clinical data or exhausting tumor tissue).

Patients’ Characteristics		Total 350 *n*. (%)	CLDN18 < 75% Tot 233 *n*. (%)	CLDN18 ≥ 75% Tot 117 *n*. (%)	*p* Value
TCGA	CIN NO	128 (36.8)	85 (24.4)	43 (12.4)	1.000
	YES	220 (63.2)	146 (42.0)	74 (21.3)	
	EBV NO	339 (97.4)	229 (65.8)	110 (31.6)	**0.0080**
	YES	9 (2.6)	2 (0.6)	7 (2.0)	
	GS NO	283 (81.3)	187 (53.7)	96 (27.6)	0.9180
	YES	65 (18.7)	44 (12.6)	21 (6.0)	
	MSI NO	294 (84.5)	192 (55.2)	102 (29.3)	0.4053
	YES	54 (15.5)	39 (11.2)	15 (4.3)	
ACRG	MSI NO	294 (84.5)	192 (55.2)	102 (29.3)	0.4053
	YES	54 (15.5)	39 (11.2)	15 (4.3)	
	MSS/EMT NO	280 (80.5)	187 (53.7)	93 (26.7)	0.8551
	YES	68 (19.5)	44 (12.6)	24 (6.9)	
	MSS/P53+ NO	227 (65.2)	145 (41.7)	82 (23.6)	0.2170
	YES	121 (34.8)	86 (24.7)	35 (10.1)	
	MSS/P53- NO	243 (69.8)	169 (48.6)	74 (21.3)	0.0752
	YES	105 (30.2)	62 (17.8)	43 (12.4)	
Birkman	EBV NO	340 (97.4)	230 (65.9)	110 (31.5)	**0.0078**
	YES	9 (2.6)	2 (0.6)	7 (2.0)	
	EBV NEG, MSS, P53 WT YES	215 (61.6)	145 (41.6)	70 (2.0)	0.7131
	NO	134 (38.4)	87 (24.9)	47 (13.5)	
	MSI NO	295 (84.5)	193 (55.3)	102 (29.2)	0.4144
	YES	54 (15.5)	39 (11.2)	15 (4.3)	
	P53 ABERRANT NO	197 (56.5)	128 (36.7)	69 (19.8)	0.5742
	YES	152 (43.6)	104 (29.8)	48 (13.8)	
Ahn	C1 NO	339 (97.4)	229 (65.8)	110 (31.6)	**0.0080**
	YES	9 (2.6)	2 (0.6)	7 (2.0)	
	C2 NO	294 (84.5)	192 (55.2)	102 (29.3)	0.4053
	YES	54 (15.5)	39 (11.2)	15 (4.3)	
	C3 NO	283 (81.3)	187 (53.7)	96 (27.6)	0.9180
	YES	65 (18.7)	44 (12.6)	21 (6.0)	
	C4 NO	230 (66.1)	146 (42.0)	84 (24.1)	0.1390
	YES	118 (33.9)	85 (24.4)	33 (9.5)	
	C5 NO	246 (70.7)	170 (48.9)	76 (21.8)	0.1218
	YES	102 (29.3)	61 (17.5)	41 (11.8)	

## Data Availability

Data are available upon request to the corresponding authors.
